# Retraction: Overexpression of the Long Noncoding RNA FOXD2-AS1 Promotes Cisplatin Resistance in Esophageal Squamous Cell Carcinoma Through the miR-195/Akt/mTOR Axis

**DOI:** 10.32604/or.2024.062048

**Published:** 2025-05-29

**Authors:** Oncology Research Editorial Office

The published article titled “Overexpression of the Long Noncoding RNA FOXD2-AS1 Promotes Cisplatin Resistance in Esophageal Squamous Cell Carcinoma Through the miR-195/Akt/mTOR Axis” has been retracted from *Oncology Research*, Vol. 28, No. 1, 2020, pp. 65–73.

DOI: 10.3727/096504019X15656904013079

URL: https://www.techscience.com/or/v28n1/48548

The authors requested this retraction, informing the Editorial Office that the experiment described in the manuscript was conducted by a third party, and issues have been encountered with the experimental process.

Following a review by the Editorial Board, the reasons provided for the retraction were found to be valid and detailed. All authors agreed to the retraction, and it was subsequently approved by the Editor-in-Chief.

As a responsible publisher, we hold the reliability and integrity of our published content in high regard. We deeply regret any inconvenience caused by this situation to our readers and all concerned parties.

